# Oral PrEP use and intention to use long-acting PrEP regimens among MSM accessing PrEP via governmental and non-governmental provision pathways, 20 European countries, October 2023 to April 2024

**DOI:** 10.2807/1560-7917.ES.2025.30.34.2500122

**Published:** 2025-08-28

**Authors:** Haoyi Wang, Alejandro Adriaque Lozano, Johann Kolstee, Hanne ML Zimmermann, Jonathan Tosh, Melanie Schroeder, Ama Appiah, Kai J Jonas

**Affiliations:** 1 Department of Work and Social Psychology, Maastricht University, Maastricht, The Netherlands; 2 Viroscience department, Erasmus Medical Centre, Rotterdam, The Netherlands; 3 ViiV Healthcare Ltd, Brentford, United Kingdom

**Keywords:** Oral PrEP use, HIV prevention, inequalities, MSM, Europe

## Abstract

**BACKGROUND:**

Pre-exposure prophylaxis (PrEP) provision routes across Europe differ notably between governmental and non-governmental pathways. The introduction of long-acting (LA)-PrEP may further diversify provision dynamics.

**AIM:**

We investigated disparities in PrEP access and whether access pathways determine oral PrEP use patterns and LA-PrEP intention among PrEP-experienced men who have sex with men (MSM).

**METHODS:**

Using data from 7,505 PrEP-experienced MSM from a cross-sectional survey (PROTECT; 20 European countries, October 2023–April 2024), we used latent class analysis (LCA) to identify MSM's latent socioeconomic positions (SEPs), and logistic regression to compare the likelihood of accessing governmental/non-governmental pathways, and compare oral PrEP adherence, discontinuation and LA-PrEP intention between governmental/non-governmental pathways.

**RESULTS:**

Most MSM accessed PrEP via governmental pathways (n = 6,671; 88.9%), 11.1% (n = 834) used non-governmental pathways. The LCA identified three groups: employed MSM with more advantaged SEPs, younger MSM with less advantaged SEPs, and older MSM with more advantaged SEPs. Compared with the first group, younger MSM with less advantaged SEPs were significantly more likely to access PrEP via non-governmental pathways (aOR = 1.27; 95% confidence interval (CI): 1.04–1.55). Accessing PrEP via non-governmental pathways was associated with suboptimal adherence (aOR = 1.28; 95% CI: 1.03–1.58), discontinuation (aOR = 3.55; 95% CI: 2.99–4.21), but also higher LA-PrEP intention (aOR = 1.28; 95% CI: 1.06–1.56).

**CONCLUSIONS:**

Inequalities exist in PrEP access among MSM in Europe. While non-governmental pathways offer opportunities to engage MSM with less advantaged SEPs, oral PrEP use patterns via this pathway were not optimal. Tailored efforts should ensure that PrEP is accessible and affordable to enhance current use and prepare for future LA-PrEP modalities.

**Key public health message**

**What did you want to address in this study and why?**

Across Europe, HIV pre-exposure prophylaxis (PrEP) provision varies notably between governmental and non-governmental pathways. However, data on usage patterns among users of non-governmental pathways are limited. We wanted to better understand how men who have sex with men (MSM) in Europe access and use PrEP, and whether their intention to use long-acting (LA)-PrEP could be determined by their current PrEP access.

**What have we learnt from this study?**

Most MSM (89%) procured PrEP through governmental and 11% via non-governmental pathways. Younger MSM, migrants and those with lower socioeconomic positions were less likely to go through governmental pathways. Users of non-governmental PrEP pathways had a 28% higher likelihood of suboptimal adherence and a threefold higher likelihood of discontinuation, yet demonstrated 28% greater intention to use LA-PrEP.

**What are the implications of your findings for public health?**

These findings highlight the inequalities in the current oral PrEP access and use patterns among MSM across Europe. Targeted efforts are needed to improve PrEP care and services within non-governmental pathways and extending those services to MSM from less advantaged backgrounds. Accessible and affordable PrEP is essential for enhancing HIV prevention and preparing for future PrEP modalities, such as LA-PrEP.

## Introduction

Pre-exposure prophylaxis (PrEP) for HIV is highly effective in preventing HIV among men who have sex with men (MSM), whether taken daily or on demand [1,2], but requires good adherence [3]. The European Centre for Disease Prevention and Control (ECDC) has recommended in 2015 that European countries integrate PrEP into their existing HIV prevention strategies for those most at risk of infection, and it was authorised by the European Medicines Agency (EMA) in 2016 [4].

Different PrEP provision pathways exist and vary across jurisdictions in Europe [5,6]. Government PrEP provision pathways are usually implemented through sexual health clinics, medical specialists or general practitioners (GP) [7]. In contrast, in countries where PrEP implementation is limited, alternative pathways are more prevalent, e.g. community-based initiatives, online pharmacies, commercial healthcare providers, or informal procurement such as using access in foreign countries while travelling, or even drug dealers [6-8]. These different access routes raise concerns about inequalities in PrEP access, particularly for MSM in less advantaged socioeconomic positions (SEPs) such as younger individuals, those with lower education or income, individuals who are unemployed and those with migration backgrounds [9-13]. Access challenges are exacerbated in countries where PrEP is not fully reimbursed [14]. While governmental pathways typically offer comprehensive services, including PrEP-related HIV and renal testing, prescriptions and monitoring, they may fail to reach all individuals even in countries with full reimbursement [15]. In some cases, MSM may opt for non-governmental pathways for reasons of convenience (e. g., waiting-lists, limited operation hours or geographically distant healthcare providers) or distrust in healthcare providers. While many governmental access pathways require no or little co-payment, this advantage is often not made use of for the aforementioned reasons. Non-governmental pathways present opportunities to engage at-risk populations missed by governmental services, offering greater convenience and autonomy, particularly in contexts where both options coexist [16,17]. Exploring non-governmental pathways as viable alternatives is therefore crucial, particularly for reaching MSM in less advantaged SEPs in contexts where governmental access is limited or PrEP reimbursement is unavailable.

Concerns have been raised about non-governmental PrEP access [18-20], particularly around suboptimal adherence and discontinuation, which can significantly compromise the effectiveness of PrEP in real-world settings on a population level [3,21,22]. Unlike governmental pathways, which follow national guidelines and are standardised, non-governmental provision involves greater uncertainty: Users may face challenges in accessing related healthcare services, obtaining prescriptions, covering costs for necessary tests and ensuring consistent medication supply [23,24]. These barriers may lead to poorer PrEP outcomes, as reliable access is crucial for adherence and persistence (i.e. continuous use) [25]. Furthermore, non-governmental PrEP use often falls outside routine PrEP surveillance and follow-up systems in Europe; hence data on usage patterns (i.e. assessment of PrEP uptake, adherence and persistence; in this study self-reported) among these users is limited. Therefore, understanding how oral PrEP use patterns differ between governmental and non-governmental provision pathways is essential for developing more tailored and effective support strategies. In this study, PrEP use patterns refer to trends in adherence and continuity of PrEP use over time, including factors such as initiation, continuation and discontinuation, rather than to specific dosing regimens.

Furthermore, novel PrEP regimens are becoming available, empowering users with greater choices and convenience in HIV prevention [26,27]. With the EMA's recent authorisation of cabotegravir as the long-acting (LA) PrEP in Europe [28], and more LA modalities in the pipeline, such as lenacapavir and islatravir [29,30], the diversity and complexity of current European PrEP provision pathways is expected to increase [31]. To date, LA-PrEP must be administered by professional healthcare providers, such as doctors, trained practitioners or nurses, because it needs to be injected [32]. This requirement limits the feasibility of LA-PrEP provision through non-governmental pathways outside healthcare settings [33]. However, as LA-PrEP has the potential to overcome challenges with adherence and persistence of oral PrEP, and to further expand the overall PrEP coverage [34,35], it is important to understand whether different oral PrEP access pathways employed by MSM would determine their interest in using alternative regimens.

In this analysis, we aimed to investigate the differences in access to PrEP provision pathways among MSM with different SEPs, and the potential variations in PrEP use adherence and discontinuation. Lastly, we aimed to investigate whether MSM’s intention to use LA-PrEP could be determined by their PrEP provision pathways.

## Methods

### Study population

We conducted a cross-sectional online survey in 20 European countries (Austria, Belgium, Cyprus, Czechia, Denmark, Finland, France, Germany, Greece, Ireland, Italy, Luxemburg, the Netherlands, Norway, Poland, Portugal, Spain, Sweden, Switzerland and the United Kingdom (UK)) from October 2023 to April 2024 (PROTECT). For this analysis, we included all the HIV-negative PrEP-experienced MSM who completed at least the 95% of our survey. The full study procedure, full survey and recruitment have been described elsewhere [36,37].

### Measures

In this analysis, among all the MSM from PROTECT, at the time of data collection, we defined their current or past PrEP provision pathways as governmental if they accessed PrEP via government PrEP programmes such as sexual health clinics/centres, medical specialists, or GPs, and as non-governmental if they accessed PrEP via other pathways such as commercial healthcare providers or online oversea pharmacies.

All outcome variables were self-reported, including oral PrEP adherence (this question was only posed to those who reported daily or on-demand oral PrEP use). For daily oral PrEP users, we asked “How many pills have you missed in the last month”, and for on-demand oral PrEP users, we asked “How many pills do you take prior to sex when you have used oral PrEP in the last 7 days and how often do you shorten the period of at least 2 h before sex”, and “How often do you forget to take one or both of the pills you must take after sexual activity (24 h and 48 h after the initial two-pill dose)”. We defined missing less than 25% of pills for daily PrEP and never missing any pills/being on time for on-demand PrEP as having optimal adherence [3]). For oral PrEP discontinuation status, we defined those who reported having discontinued oral PrEP and currently not using oral PrEP at the time of data collection as discontinued oral PrEP, without considering temporal pausing due to HIV seroconversion or medical contraindications. We measured LA-PrEP intention on a 5‐point Likert scale ranging from extremely unwilling (1) to extremely willing (5). In this study, we dichotomised LA-PrEP intention as either higher intention (extremely willing (5) or somehow willing (4) or lower intention (the rest of the scale points (1–3)).

The SEP variables included education attainment, employment status and perceived financial status, following the definition from the American Psychological Association [38]. However, given its close associations with age and migration background [39,40], and because age and migration background were also closely associated with PrEP use [13], we included age and migration background (first- or second-generation migrants, based on their country of birth and country of residence) as SEP variables in this analysis.

**Figure 1:**
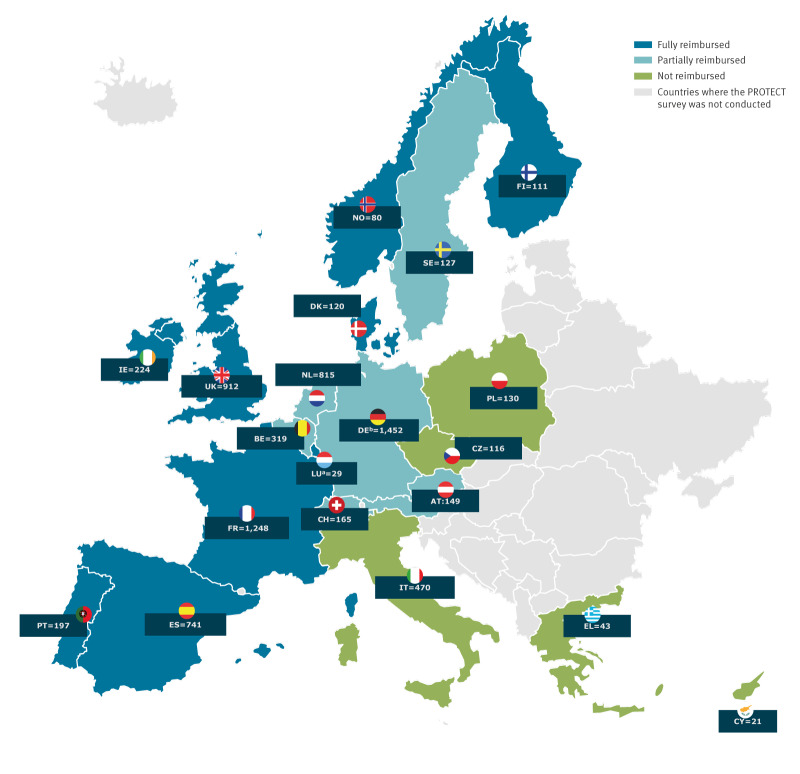
Country of residence of PrEP-experienced MSM participants from the PROTECT Study in 20 European countries, with country classifications for PrEP reimbursement status, October 2023–April 2024 (n = 7,505)

AT: Austria; BE: Belgium; CH: Switzerland; CY: Cyprus; CZ: Czechia; DE: Germany; DK: Denmark; ES: Spain; FI: Finland; FR: France; EL: Greece; IE: Ireland; IT: Italy; LU: Luxembourg; MSM: men who have sex with men; NL: the Netherlands; NO: Norway; PL: Poland; PrEP: pre-exposure prophylaxis; PT: Portugal; SE: Sweden; UK: United Kingdom.

To adjust for the potential impact of the availability and affordability of PrEP access via governmental provision pathways, the reimbursement status of oral PrEP was defined based on the participant’s country of residence. We considered a country with a fully-reimbursed status as one in which people can access PrEP without paying a copayment, a country with partly-reimbursed status as one in which people can access PrEP with copayment, and a country with non-reimbursed status as one in which people can only access PrEP out of pocket. Our classification of countries into these categories (Figure 1) was based on policy settings up to and including 1 January 2023, given that our data were collected in October 2023, and assuming that our results would not be impacted by study contexts such as in Italy where oral PrEP was recently made available, at a time partly overlapping with our data collection [41].

Fully reimbursed: countries where the PROTECT survey was conducted and people can access oral PrEP without paying a copayment; partially reimbursed: countries where the PROTECT survey was conducted and people can access oral PrEP with copayment assigned; not reimbursed: countries where the PROTECT survey was conducted and people can only access oral PrEP out-of-pocket. The numbers in the boxes represent the number of participants included in each country in this analysis.

### Statistical analysis

#### Descriptive analysis

We first used descriptive statistical analyses to define our participants’ SEP variables, and oral PrEP reimbursement status relative to their oral PrEP access pathways (governmental/non-governmental). Similarly, we described our outcome variables among the overall sample. A chi-squared test was performed to compare the differences between these groups for each variable. To support country-/region-level decision-making, we further described our outcome variables by country of residence if that country had more than 500 survey responses from PrEP-experienced MSM to ensure sufficient statistical power: France, Germany, the Netherlands, Spain and the UK. For sensitivity purposes, we also described our outcome variables for the rest of the countries with smaller sample sizes that had fewer than 500 survey responses from PrEP-experienced MSM.

#### Latent class analysis

We performed a latent class analysis (LCA) to classify the latent SEP profiles of our participants, including all SEP variables. The LCA is a statistical approach that generates latent classes based on multivariate categorical data patterns to group similar individuals, without pre-defining classes’ characteristics or any assumptions [42]. To improve public health applicability and clinical practice by minimising the number of the estimated latent classes, were tested a series of LCA models with one to six classes. To avoid the likelihood of converging on a local maximum, 500 start values were generated for each model. We used the Akaike information criterion (AIC), and sample size-adjusted Bayesian Information criterion (BIC) to assess the model’s goodness of fit. We selected the final model based on the AIC and BIC values. We then described the SEP characteristics to define the identified latent SEP backgrounds.

#### Logistic regression analyses

To explore the associations between the latent SEP backgrounds and their likelihood of accessing different PrEP provision pathways, we conducted a multivariable logistic regression, adjusting for the oral PrEP reimbursement status given its close association with oral PrEP uptake via governmental PrEP provision pathways, regardless of their significance. Then, to explore the variations in oral PrEP use patterns across different PrEP provision pathways, we conducted multivariable logistic regression analyses to access the associations between the PrEP provision pathways and oral PrEP (i) suboptimal adherence, (ii) discontinuation and (iii) LA-PrEP intention, adjusting all for their latent SEP profiles and the oral PrEP reimbursement status. Regression diagnostics for all regression analyses did not reveal collinearity, conspicuous values or normality violations. We considered p values < 0.05 as significant. All analyses were conducted in R (version R 4.3.2). For the LCA in this analysis, we used the poLCA package (v1.6.0.1).

## Results

### Study population characteristics

Of the 15,458 MSM survey responses collected from the PROTECT survey, all 7,505 HIV-negative PrEP-experienced MSM were included in this analysis. Of these, 6,671 (88.9%) accessed PrEP via governmental PrEP provision pathways, and 834 (11.1%) accessed via non-governmental PrEP provision pathways.

Overall, the median age in the total sample was 40 years (P25–P75: 33–48). The majority (45.8%) were living in countries where PrEP was fully reimbursed, followed by 40.3% living in countries where PrEP was partially reimbursed, and only a minority (13.9%) were living in countries where PrEP was not reimbursed for the governmental PrEP provision pathways. Compared with MSM accessing oral PrEP via governmental pathways, MSM who accessed oral PrEP via non-governmental pathways were less likely to be employed, more likely to report struggling with current income, more likely to be a migrant, and also more likely to be living in a country where oral PrEP was fully reimbursed (Figure 1). For detailed SEP characteristics among the overall sample, see Table 1.

**Table 1:** Study socioeconomic position characteristics of PrEP users by provision pathway, 20 European countries, October 2023–April 2024 (n = 7,505)

Variable	Governmental pathways (n = 6,671)		Non-governmental pathways (n = 834)		Total sample (n = 7,505)		
	n	%	n	%	N	%	p value
Age (years)							
Median (P25–P75)	40	33–47	41	33–49	40	33–48	0.384
18–24	276	4.1	42	5.0	318	4.2	
25–29	719	10.8	91	10.9	810	10.8	
30–39	2,298	34.4	239	28.7	2,537	33.8	
40–49	1,854	27.8	247	29.6	2,101	28.0	
50–59	984	14.8	137	16.4	1,121	14.9	
60–69	281	4.2	36	4.3	317	4.2	
≥ 70	259	3.9	42	5.0	301	4.0	
Education							
I do not have a high school diploma	105	1.6	15	1.8	120	1.6	0.512
Secondary education (high school or equivalent)	1,542	23.1	187	22.4	1,729	23.0	
Bachelor’s degree (university or equivalent)	2,098	31.4	263	31.5	2,361	31.5	
Master’s degree (university or equivalent)	2,478	37.1	300	36.0	2,778	37.0	
PhD / Doctorate	448	6.7	69	8.3	517	6.9	
Employment							
Employed	5,447	81.7	651	78.1	6,098	81.3	0.029
Other	392	5.9	48	5.8	440	5.9	
Retired/medical leave	221	3.3	32	3.8	253	3.4	
Student	350	5.2	54	6.5	404	5.4	
Unemployed	261	3.9	49	5.9	310	4.1	
Perceived income							
Living really comfortably on present income	589	8.8	61	7.3	650	8.7	0.006
Living comfortably on present income	2,600	39.0	361	43.3	2,961	39.5	
Neither comfortable nor struggling on present income	2,290	34.3	275	33.0	2,565	34.2	
Struggling on present income	855	12.8	82	9.8	937	12.5	
Really struggling on present income	337	5.1	55	6.6	392	5.2	
Migration background							
Non-migrant	4,219	63.2	572	68.6	4,791	63.8	< 0.001
First-generation migrant	1,840	27.6	177	21.2	2,017	26.9	
Second-generation migrant	612	9.2	85	10.2	697	9.3	
Oral PrEP reimbursement status							
Fully reimbursed^a^	2,891	43.3	547	65.6	3,438	45.8	< 0.001
Partially reimbursed^b^	2,860	42.9	167	20.0	3,027	40.3	
Not reimbursed^c^	920	13.8	120	14.4	1,040	13.9	

PrEP: pre-exposure prophylaxis.

^a^ People can access PrEP without paying a copayment.

^b^ People can access PrEP with copayment was assigned.

^c^ People can only access PrEP out-of-pocket.

### Oral PrEP use patterns and LA PrEP intention

**Figure 2:**
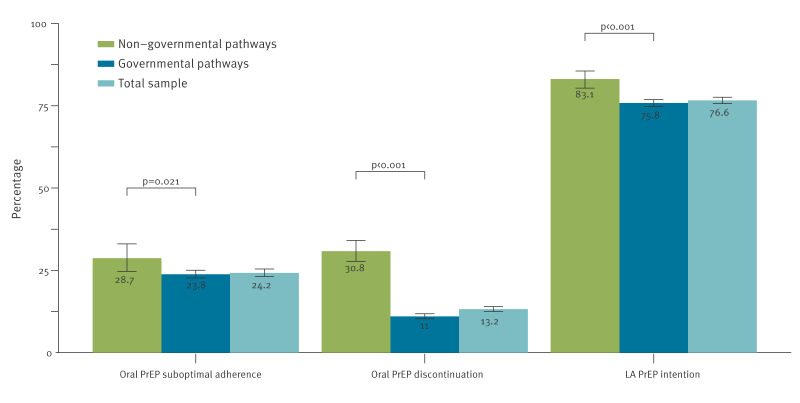
Oral PrEP use patterns and long-acting PrEP use intention between MSM accessing PrEP via governmental and non-governmental PrEP provision pathways, 20 European countries, October 2023–April 2024 (n = 7,505)

LA: long-acting; MSM: men who have sex with men; PrEP: pre-exposure prophylaxis.

Figure 2 summarises oral PrEP adherence, oral PrEP discontinuation and LA-PrEP intention by oral PrEP provision pathways. Overall, 24.2% (95% confidence interval (CI): 23.12–25.40) reported suboptimal adherence, 13.2% (95% CI: 12.5–14.0) reported discontinuing using oral PrEP, and 76.6% (95% CI: 75.6–77.6) showed a high intention to use LA-PrEP if it becomes available and accessible. Among MSM who accessed oral PrEP via governmental pathways, 23.8% (95% CI: 22.7–25.0) reported suboptimal adherence, 11.0% (95% CI: 10.3–11.8) reported discontinuing using oral PrEP, and 75.8% (95% CI: 74.8–76.8) showed a high intention to use LA-PrEP. Conversely, MSM who accessed oral PrEP via non-governmental pathways reported higher oral PrEP suboptimal adherence (28.7%; 95% CI: 24.7–33.0; p = 0.021), higher oral PrEP discontinuation (30.8%; 95% CI: 27.7–34.1; p < 0.001), and higher LA-PrEP intention (83.1%; 95% CI: 80.3–85.5; p < 0.001). These pan-European trends were mirrored on the country level; we append the results for the individual countries in Supplementary Figures S1–S6.

Differences between different PrEP provision pathways were tested by chi-squared test. LA-PrEP intention was measured on a 1–5 Likert scale; we considered having extreme/somehow intention as having a high LA-PrEP intention in this analysis. Error bars show 95% confidence intervals using continuity correction methods.

### Latent class analysis

A comparison of model fit indices demonstrated that a three-class latent SEP background was preferable; a comparison of latent class models with 1–6 classes fit indices, including maximum log likelihoods, AICs and BICs, is appended in Supplementary Table S1. Although the three-class model did not have the smallest AIC (86,519.4) and the smallest BIC (86,948.6), the model’s AIC and BIC improvement diminished substantially beyond three classes, indicating that a model with four classes or more only led to marginal improvement compared with a model of three classes in terms of the statical indices. We therefore selected the three-class solution as it optimally balanced statistical fit and practical interpretability for public health implementation.

**Figure 3:**
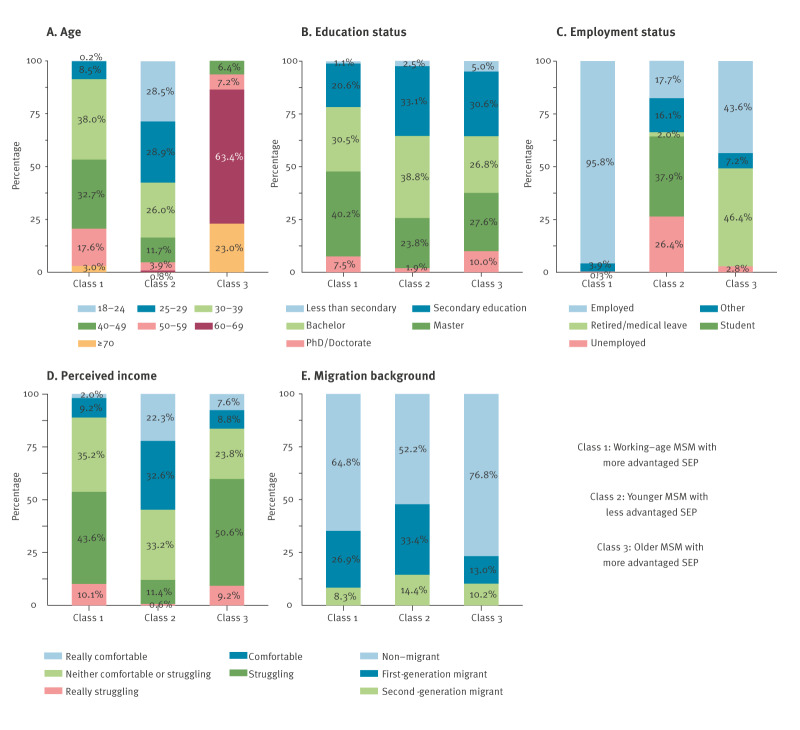
Socioeconomic position characteristics across identified three-class latent socioeconomic position backgrounds, 20 European countries, October 2023–April 2024 (n = 7,505)

MSM: men who have sex with men; SEP: socioeconomic position.

As shown in Figure 3, Class 2 (n = 1,065; 14.2%) had the least advantaged latent SEP background. The MSM in this class were the youngest, least educated and least employed, and were most likely to be students. This class also had the highest proportion of individuals who perceived themselves to be struggling with their current income, and it had the highest proportion of migrants (hereafter called younger MSM with less advantaged SEPs). Members of Class 1 (n = 5,940; 79.1%) and Class 3 (n = 500; 6.7%) had similar levels of education and perceived income. However, major differences existed in their age and employment status. The MSM in Class 3 were notably older, with 86% of individuals older than 60 years, and a higher proportion was retired or on medical leave, typically associated with older age. Class 1 comprised exclusively employed individuals (96%), reflecting working-age adults in Europe. This indicates a similar SEP background in Class 1 (working-age MSM with more advantaged SEPs) and Class 3 (older MSM with more advantaged SEPs), with a major distinction based on age.

For detailed percentages per SEP characteristics, see Supplementary Table S2.

### Differences in accessing governmental and non-governmental PrEP provision pathways

Table 2 outlines detailed information on the multivariable logistic regressions that investigated the association between the latent SEP backgrounds and their likelihood of accessing different PrEP provision pathways (Model 1). There were no significant differences (adjusted odds ratio (aOR) = 1.28; 95% CI: 0.96–1.68) in accessing oral PrEP between working-age MSM and older MSM with more advantaged SEP However, compared with working-age MSM with more advantaged SEP, the younger MSM with less advantaged SEP were significantly more likely to access their oral PrEP via non-governmental pathways (aOR = 1.27; 95% CI: 1.04–1.55).

**Table 2:** Logistic regression models among PrEP-experienced MSM, 20 European countries, October 2023–April 2024 (n = 7,505)

Covariate		aOR	95% CI	p value
Model 1: Latent SEP backgrounds and the likelihood of accessing non-governmental PrEP provision pathways				
Latent socioeconomic position background	Class 1^a^	Reference		
	Class 2^b^	1.27	1.04–1.55	0.018
	Class 3^c^	1.28	0.96–1.68	0.079
Oral PrEP reimbursement status	Fully reimbursed	Reference		
	Partially reimbursed	0.31	0.26–0.37	< 0.001
	Not reimbursed	0.69	0.56–0.85	0.001
Model 2: PrEP provision pathways and the likelihood of suboptimal oral PrEP adherence				
PrEP provision pathway	Governmental	Reference		
	Non-governmental	1.28	1.03–1.58	0.022
Latent socioeconomic position background	Class 1^a^	Reference		
	Class 2^b^	1.34	1.12–1.59	0.001
	Class 3^c^	0.60	0.45–0.78	< 0.001
Oral PrEP reimbursement status	Fully reimbursed	Reference		
	Partially reimbursed	0.95	0.83–1.08	0.442
	Not reimbursed	0.91	0.74–1.10	0.326
Model 3: PrEP provision pathways and the likelihood of discontinuing oral PrEP				
PrEP provision pathway	Governmental	Reference		
	Non-governmental	3.55	2.99–4.21	< 0.001
Latent socioeconomic position background	Class 1^a^	Reference		
	Class 2^b^	1.90	1.60–2.25	< 0.001
	Class 3^c^	0.83	0.60–1.11	0.219
Oral PrEP reimbursement status	Fully reimbursed	Reference		
	Partially reimbursed	0.95	0.81–1.11	0.515
	Not reimbursed	1.62	1.34–1.95	< 0.001
Model 4: PrEP provision pathways and the likelihood of having a high LA-PrEP intention				
PrEP provision pathway	Governmental	Reference		
	Non-governmental	1.28	1.06–1.56	0.011
Latent socioeconomic position background	Class 1^a^	Reference		
	Class 2^b^	1.04	0.89–1.22	0.663
	Class 3^c^	0.81	0.66–1.00	0.050
Oral PrEP reimbursement status	Fully reimbursed	Reference		
	Partially reimbursed	0.42	0.37–0.47	< 0.001
	Not reimbursed	0.79	0.66–0.95	0.009

aOR: adjusted odds ratio; CI: confident intervals; MSM: men who have sex with men; PrEP: pre-exposure prophylaxis; SEP: socioeconomic position.

^a^ Working-age MSM with more advantaged socioeconomic positions.

^b^ Younger MSM with less advantaged socioeconomic positions.

^c^ Older MSM with more advantaged socioeconomic positions.

Model 1: The variance inflation factors for these variables ranged from 1.002 to 1.003, suggesting negligible multi-collinearity. Model 2: The variance inflation factors for these variables ranged from 1.001 to 1.025, suggesting negligible multi-collinearity. Model 3: The variance inflation factors for these variables ranged from 1.003 to 1.041, suggesting negligible multi-collinearity. Model 4: The variance inflation factors for these variables ranged from 1.003 to 1.023, suggesting negligible multi-collinearity.

In addition, oral PrEP reimbursement via governmental pathways also played a significant role. Compared with MSM living in a country where oral PrEP was fully reimbursed via their governmental PrEP provision pathways, MSM who lived in a country where oral PrEP was partially reimbursed (aOR = 0.31; 95% CI: 0.26–0.37) and not reimbursed (aOR = 0.69; 95% CI: 0.56–0.78) were less likely to access their oral PrEP via non-governmental pathways.

### Variations in oral PrEP use patterns between governmental and non-governmental PrEP provision pathways

Table 2 further outlines detailed information on the multivariable logistic regressions investigating the associations between the different PrEP provision pathways and oral PrEP use suboptimal adherence (Models 2) and discontinuation (Model 3).

For suboptimal oral PrEP adherence as the endpoint, MSM who accessed their PrEP via non-governmental pathways showed a significantly higher likelihood of having suboptimal adherence (aOR = 1.28; 95% CI: 1.03–1.58). Compared with the working-age MSM with more advantaged SEP backgrounds, the older MSM with more advantaged SEP backgrounds were less likely to show suboptimal adherence (aOR = 0.60; 95% CI: 0.45–0.78), while the younger MSM with less advantaged SEP backgrounds were more likely to show suboptimal adherence (aOR = 1.34; 95% CI: 1.12–1.59).

For oral PrEP discontinuation as the endpoint, MSM who accessed their PrEP via non-governmental pathways similarly showed a significantly higher likelihood of suboptimal adherence (aOR = 3.55; 95% CI: 2.99–4.21). No differences were found between the working-age and older MSM with more advantaged SEP backgrounds. However, the younger MSM with less advantaged SEP backgrounds were more likely to discontinue using oral PrEP (aOR = 1.90; 95% CI: 1.60–2.25). Yet, MSM who lived in a country where oral PrEP was not reimbursed were significantly more likely to discontinue oral PrEP (aOR = 1.62; 95% CI: 1.34–1.95).

### Intention to use long-acting PrEP between governmental and non-governmental PrEP provision pathways

Table 2 also outlines the multivariable logistic regressions investigating the association between the different PrEP provision pathways and LA-PrEP intention. Compared with MSM who accessed their PrEP via governmental pathways, users via non-governmental pathways showed a higher likelihood of a high LA-PrEP intention (aOR = 1.28; 95% CI: 1.06–1.56). No differences were found between the working-age MSM with more advantaged SEP backgrounds (Class 1) and the younger MSM with less advantaged SEP backgrounds. However, older MSM with more advantaged SEP backgrounds showed a borderline significance in a lower intention (aOR = 0.81; 95% CI: 0.66–1.00). In addition, compared with MSM living in a country where oral PrEP was fully-reimbursed, MSM who lived in a country where oral PrEP was partially-reimbursed (aOR = 0.42; 95% CI: 0.37–0.47) or not reimbursed (aOR = 0.79; 95% CI: 0.66–0.95) were less likely to have a higher intention to use LA-PrEP.

## Discussion

In Europe, non-governmental pathways outside the government PrEP programmes provide additional access opportunities to reach MSM who are younger and have less advantaged SEPs and who are less likely to engage with governmental services—particularly in countries where oral PrEP is fully reimbursed. However, we observed less optimal oral PrEP adherence and persistence among users in these non-governmental pathways, indicating gaps in PrEP provision services between governmental and non-governmental pathways across Europe in this analysis.

Our findings are particularly relevant since most PrEP surveillance systems fail to reflect the PrEP services gaps between different PrEP provision pathways. In European countries where PrEP is relatively well implemented, such as France where national PrEP delivery is centralised [11], or the Netherlands where the national PrEP programme is decentralised but heavily reliant on sexual health clinics as the sole PrEP provider [23], the monitoring of PrEP use often excludes users accessing PrEP via other pathways. Consequently, the invisibility of non-governmental PrEP use bars the delivery and the access to interventions aimed at improving PrEP adherence and persistence [43]. In European countries where PrEP implementation is limited and non-governmental pathways are the primary means of access, such as Greece or Cyprus [8], there has not been any surveillance or research focusing on PrEP use qualities or patterns. This lack of data exacerbates gaps and potential missed opportunities in HIV prevention [44]. 

Our study found that non-governmental pathways were not only more often accessed by MSM with less advantaged SEPs, but their users also had less optimal adherence and persistence. One reason may be the limited and less optimal PrEP services available in these non-governmental pathways [18,19]. The prerequisite for adherence and persistence is access [25]. In the Netherlands, the national PrEP programme has very limited provision capacities, resulting in a long waiting list for governmental pathway access, and many MSM are currently accessing PrEP via alternative PrEP providers [23]. However, even healthcare providers, such as GPs, within the governmental pathways in the Netherlands often show reluctance to prescribe PrEP [24,45]. This reluctance—combined with the lack of structured support in non-governmental pathways—may further contribute to challenges among MSM accessing PrEP via non-governmental pathways in maintaining stable PrEP prescription renewals, ultimately affecting adherence and persistence. In countries where PrEP implementation is limited, such as Cyprus, MSM must rely on online pharmacies from overseas to access PrEP [8]. The unstable medication supply from these sources further disrupts consistent adherence and persistence. Another reason for suboptimal adherence among MSM using non-governmental pathways may be the users’ less advantaged SEP, as revealed in this analysis. These findings align with previous meta-analyses investigating determinants of PrEP adherence and discontinuation [21,22]. Therefore, while encouraging the non-governmental pathways to offer opportunities for less advantaged MSM, it is also important that public health authorities ensure improved services and reliable medication access within non-governmental pathways.

Our study also found that MSM who accessed oral PrEP via non-governmental pathways showed a higher LA-PrEP intention compared with those who use governmental pathways. However, although LA-PrEP could potentially address the adherence and persistence challenges often seen in non-governmental pathways, it will only be accessible through governmental pathways where healthcare linkage is better guaranteed [46,47]. Consequently, despite a higher intention for LA-PrEP among non-governmental pathway users, they will probably not be able to access and benefit from it through their current PrEP access pathways. Therefore, users of non-governmental pathways may be under-reached and under-served for LA-PrEP when it becomes available. To maximise the public health impact of LA-PrEP, it is essential to ensure equal access across different PrEP provision pathways in Europe and to improve healthcare linkage for all PrEP users [46,47]. We further stress that equitable LA-PrEP access will require comprehensive strategies encompassing medication regulation policies, pricing, reimbursement levels and coordinated funding mechanisms [48-50], including reaching MSM with less advantaged socioeconomic backgrounds who currently rely on non-governmental pathways.

We found that MSM residing in countries where oral PrEP is not fully reimbursed were less likely to access PrEP via non-governmental pathways. This suggests that cost-driven structural barriers continue to impact oral PrEP access in Europe, as the high cost of oral PrEP is likely to reduce its uptake [51]. In other words, given a relatively higher copayment of oral PrEP from the governmental access, people may expect even higher costs from the non-governmental access [23], resulting in lower PrEP uptake among non-governmental pathways. In addition, non-reimbursed oral PrEP status determined discontinuation but not adherence, reinforcing our suggestion that cost affects the continuous uptake of oral PrEP. Therefore, it is crucial not only to make oral PrEP available and accessible but also to ensure it is affordability, regardless of the method of procurement. Interestingly, we found that MSM in countries where oral PrEP is not fully reimbursed had lower LA-PrEP intention. This indicates the importance of and the higher demand for oral PrEP as an HIV prevention modality among MSM in these countries. It also underscores the importance of comprehensive oral PrEP implementation before the introduction of LA-PrEP in countries where oral PrEP is not yet fully reimbursed.

Our study has some limitations. Firstly, our sample may underrepresent individuals accessing oral PrEP through non-governmental pathways, as only 10% of participants fell into this category. This underrepresentation may be partly due to the online recruitment strategy of the PROTECT study, which inherently limits participation to individuals with Internet access. As a result, our sample may be skewed towards individuals of higher socioeconomic status, potentially overlooking more marginalised MSM populations who are less likely to participate in internet-based surveys and more likely to access PrEP through informal or non-governmental pathways. This imbalance may have introduced statistical limitations, such as floor or ceiling effects, and reduced power to detect meaningful differences within the non-governmental subgroup. Nevertheless, the significant differences observed between participants accessing PrEP via governmental vs non-governmental pathways suggest that our findings are still relevant and valid, although they should be interpreted with caution. Another limitation of our analysis can be the smaller sample sizes from other countries other than France, Germany, the Netherlands, Spain and the UK, especially from Eastern Europe. Consequently, we did not have sufficient power to conduct any within-country comparison for these countries between governmental and non-governmental pathways on a country level. However, given the similar findings we identified among the countries with smaller sample sizes (see Supplementary Figure S6), we believe our findings are not limited to the larger-sample countries. We therefore consider our results remain valid for pan-European estimates. Secondly, our definitions of governmental and non-governmental pathways may not account for all specific variations across countries. Some pathways may be considered governmental in certain jurisdictions but were categorised as non-governmental in our study (e.g. a doctor volunteering to administer PrEP in a community health centre), or vice versa, potentially biasing our findings and limiting our interpretation of PrEP provision pathways. Similarly, the cross-sectional nature of our data prevents longitudinal assessment, making our findings time-sensitive and unable to establish causal relationships. While we identified associations between non-governmental pathway use and suboptimal PrEP adherence/discontinuation, we cannot determine whether these reflect true differences or whether individuals with adherence challenges preferentially seek alternative access routes, or if there were other structural cofounders such as general economic and cultural differences in the included countries. These limitations underscore the need for longitudinal, real-world studies to better characterise differences between PrEP provision pathways and their clinical implications. Lastly, we did not include data on the re-initiation of oral PrEP among MSM, potential switching between PrEP provision pathways, or the existence of mixed pathways. These aspects were beyond the scope of our study and should be addressed in future research.

## Conclusions

Our findings highlighted differences in accessing PrEP provision pathways and correlated endpoints among MSM across Europe. We stress and recommend that future PrEP services and monitoring also start to include users from the non-governmental pathways, making sure that all PrEP users can be supported. In the meantime, it is also important for the European public health authorities to ensure improved services and reliable medication access within non-governmental pathways to better support MSM with less advantaged SEP backgrounds. To achieve that, ensuring a stable PrEP medication supply and robust healthcare linkage for everyone is particularly crucial. In addition, making PrEP accessible and affordable is essential for enhancing the PrEP use cascade and preparing for future PrEP modalities, such as LA-PrEP.

## Data Availability

Data are available upon request from the corresponding author.

## Supplementary Data

834000 Supplement
