# Authors’ correction for Euro Surveill. 2025;30(34)

**DOI:** 10.2807/1560-7917.ES.2025.30.38.250925c

**Published:** 2025-09-25

**Authors:** Eurosurveillance editorial team

**Affiliations:** 1 European Centre for Disease Prevention and Control (ECDC), Stockholm, Sweden

In the article ‘Oral PrEP use and intention to use long-acting PrEP regimens among MSM accessing PrEP via governmental and non-governmental provision pathways, 20 European countries, October 2023 to April 2024’ by Wang et al. published in *Eurosurveillance* on 28 August 2025, the percentages in Figure 3, Panel D were wrong at the time of publication. Panel D in Figure 3 and Supplementary Table S2, where detailed percentages are displayed, were corrected and replaced upon the authors' request on 24 September 2025.

