# Supplementary material for: Oral PrEP use and intention to use long-acting PrEP regimens among MSM accessing PrEP via governmental and non-governmental provision pathways, 20 European countries, October 2023 to April 2024
Source: Euro Surveill. 2025 Aug 28;30(34):2500122. doi: 10.2807/1560-7917.ES.2025.30.34.2500122 (PMC12397725; doi:10.2807/1560-7917.ES.2025.30.34.2500122)
Supplement: Supplement [file 25-00122_WANG_Supplement.pdf]

Supplementary materials

"This supplementary material is hosted by *Eurosurveillance* as supporting information alongside the article [Oral PrEP use and the intention to use long-acting PrEP regimens among MSM accessing PrEP via governmental and non-governmental provision pathways in 20 European Countries, October 2023 – April 2024], on behalf of the authors, who remain responsible for the accuracy and appropriateness of the content. The same standards for ethics, copyright, attributions and permissions as for the article apply. Supplements are not edited by *Eurosurveillance* and the journal is not responsible for the maintenance of any links or email addresses provided therein."

Supplementary Figures

Figure S1. Oral PrEP use patterns and long-acting PrEP use intention between MSM accessing PrEP via governmental and non-governmental PrEP provision pathways in France, October 2023–April 2024 (n = 1,248)

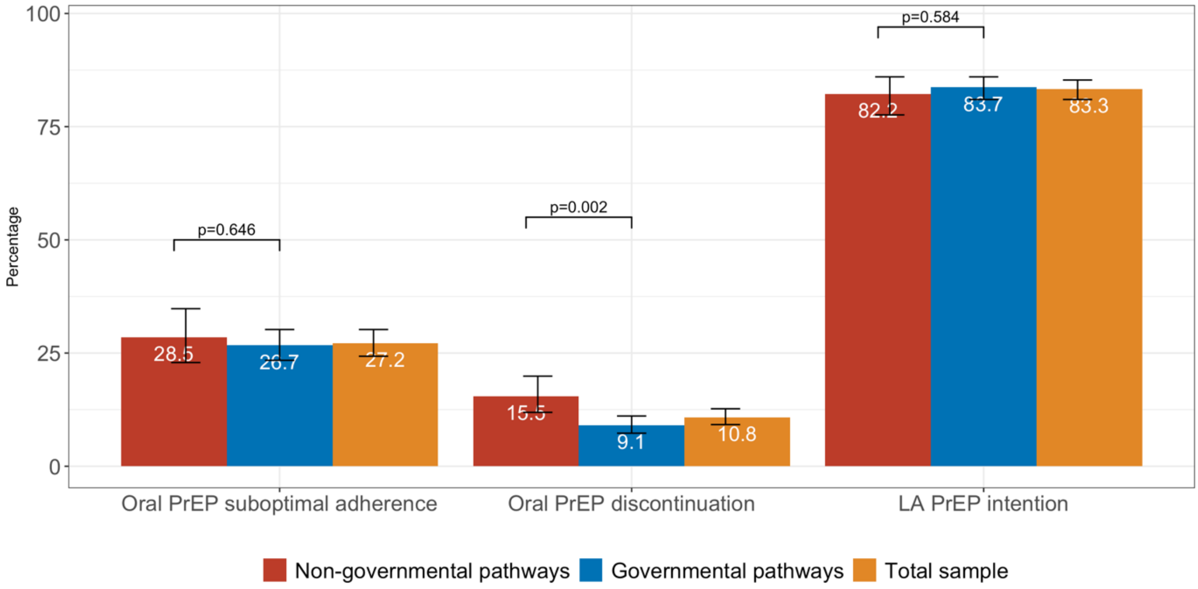

Figure S2. Oral PrEP use patterns and long-acting PrEP use intention between MSM accessing PrEP via governmental and non-governmental PrEP provision pathways in Germany, October 2023–April 2024 (n = 1,452)

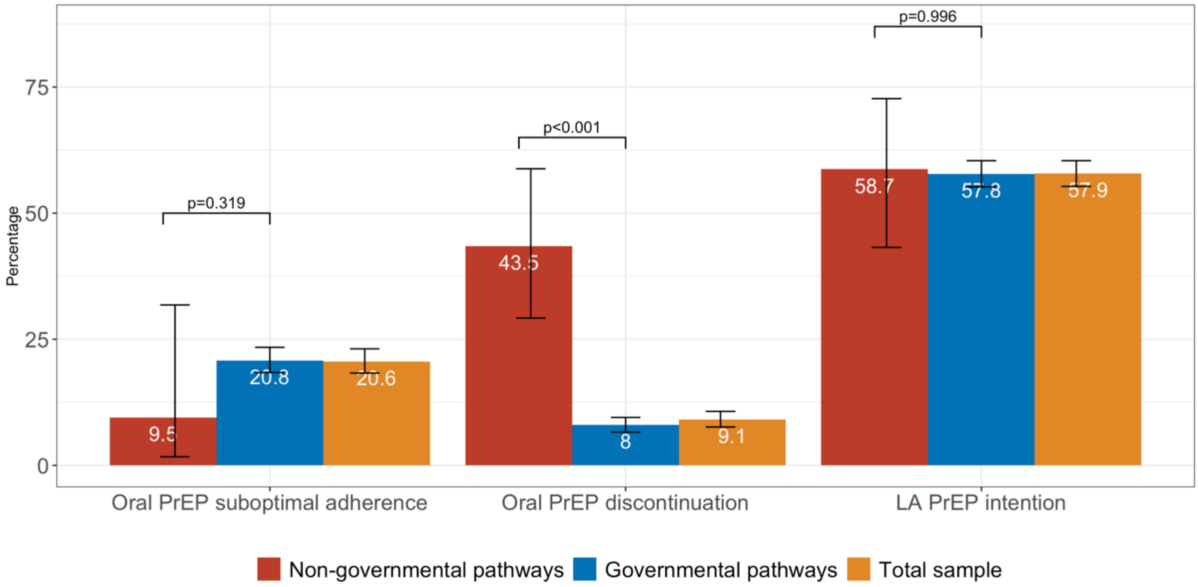

Figure S3. Oral PrEP use patterns and long-acting PrEP use intention between MSM accessing PrEP via governmental and non-governmental PrEP provision pathways in the Netherlands, October 2023–April 2024 (n = 815)

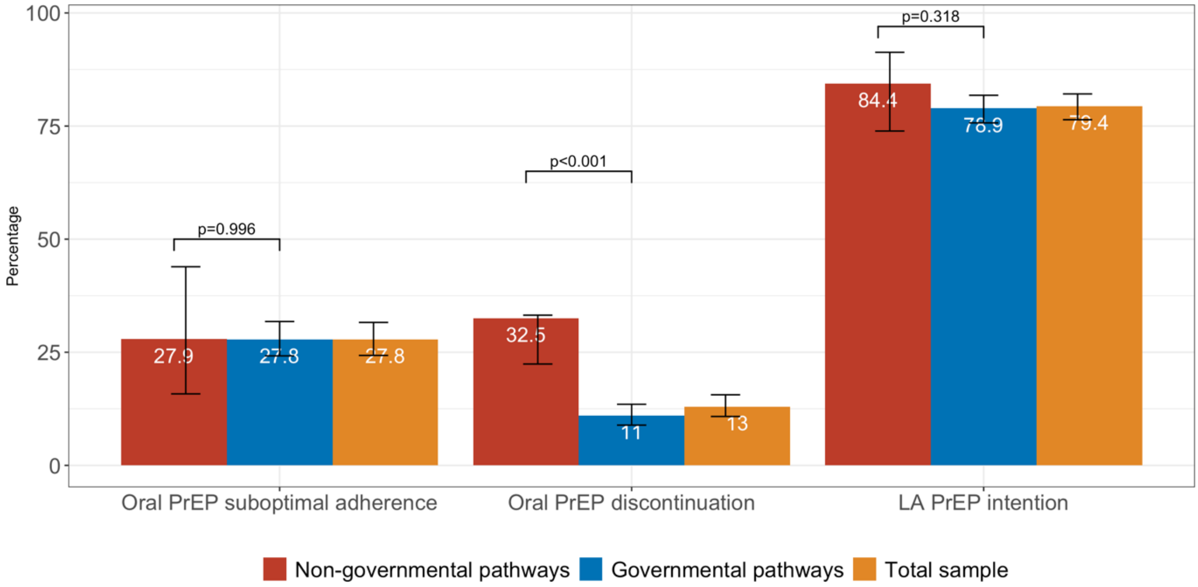

Figure S4. Oral PrEP use patterns and long-acting PrEP use intention between MSM accessing PrEP via governmental and non-governmental PrEP provision pathways in Spain, October 2023–April 2024 (n = 741)

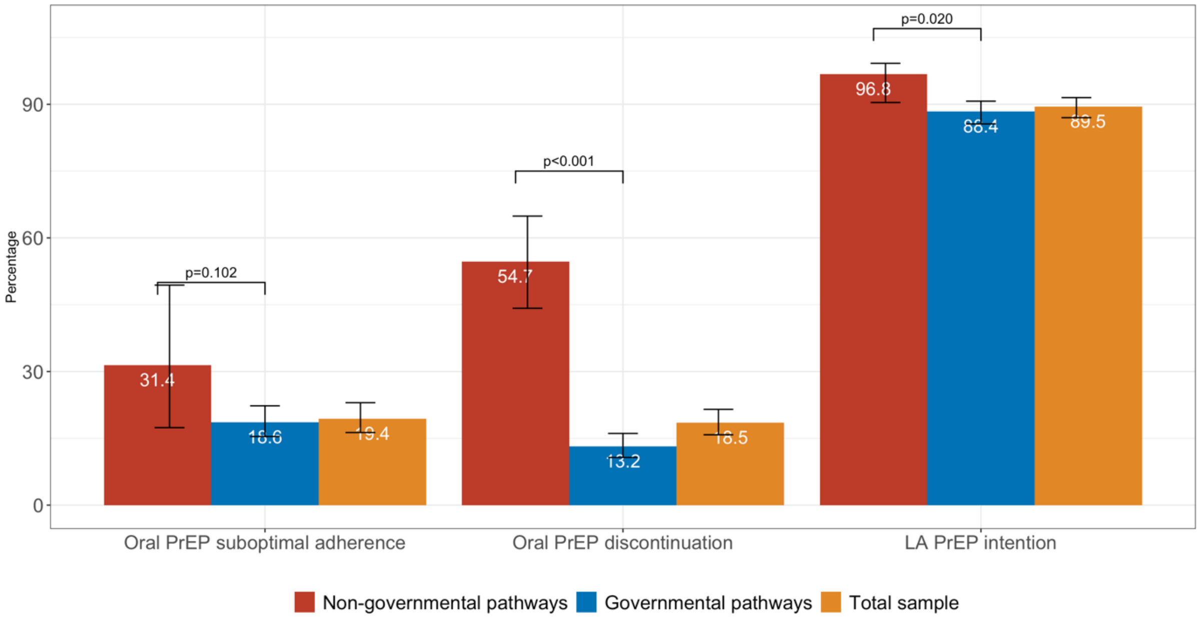

Figure S5. Oral PrEP use patterns and long-acting PrEP use intention between MSM accessing PrEP via governmental and non-governmental PrEP provision pathways in the United Kingdom, October 2023–April 2024 (n = 912)

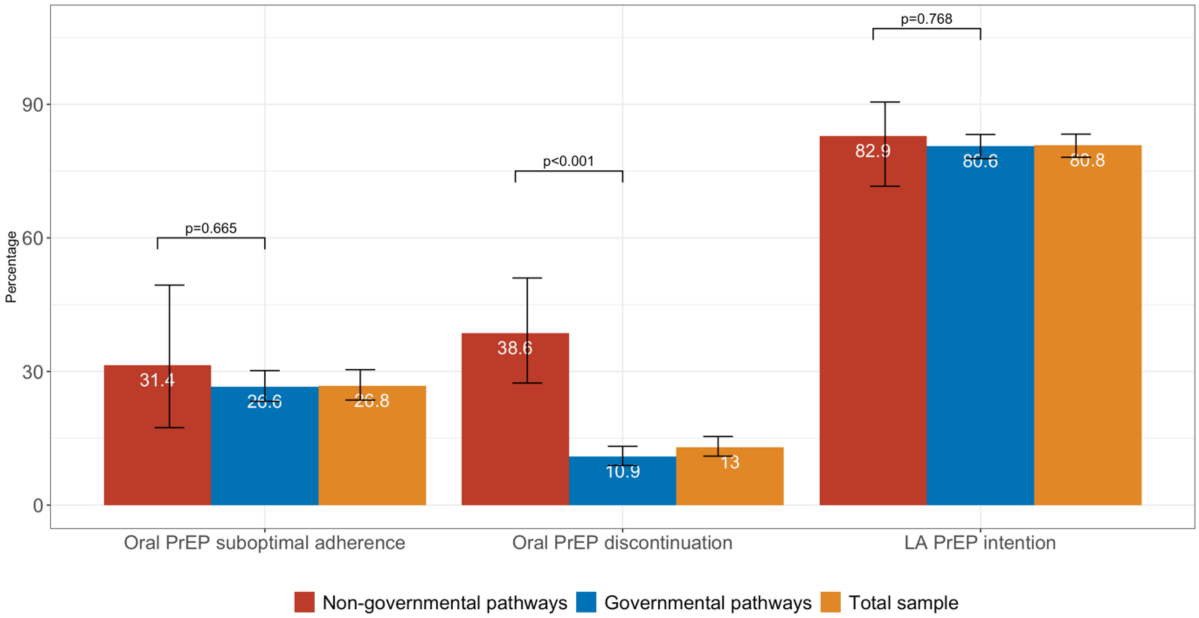

Figure S6. Oral PrEP use patterns and long-acting PrEP use intention between MSM accessing PrEP via governmental and non-governmental PrEP provision pathways in the rest 15 European countries, October 2023–April 2024 (n = 2,337)

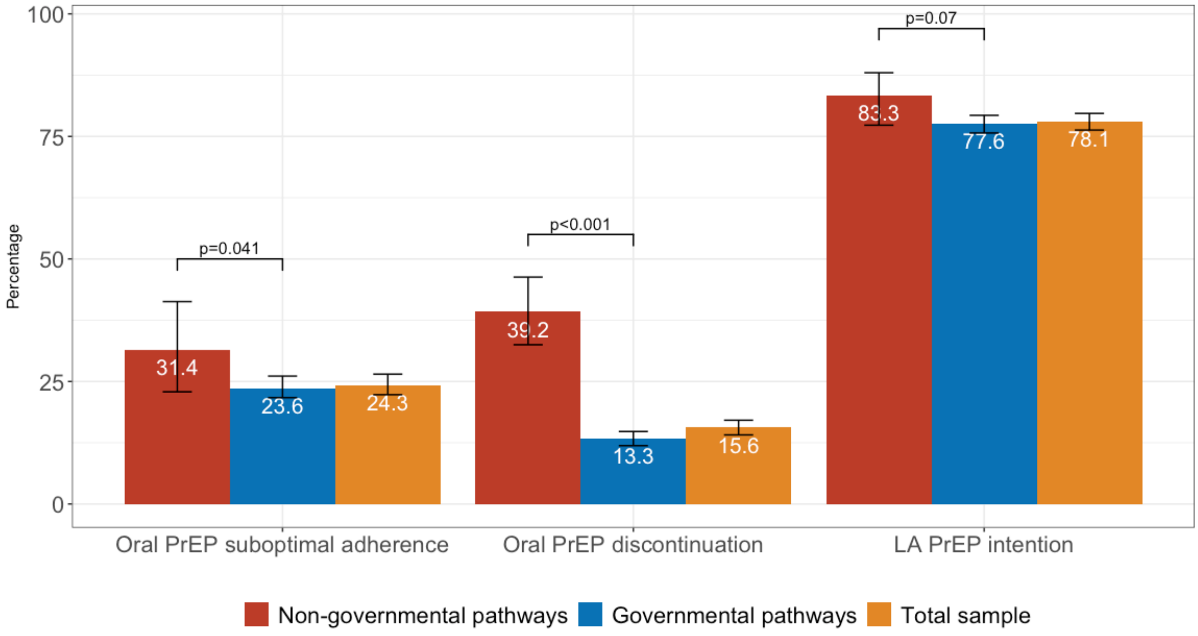

Table S1. Latent class analysis fit indices for 1-6 class model

Model	Maximum log likelihood (# parameters)	AIC	BIC
1 Class	-44448.4 (20)	88936.7	89075.2
2 Classes	-43597.2 (41)	87272.4	87560.2
3 Classes	-43197.7 (62)	86519.4	86948.6
4 Classes	-42924.2 (83)	86434.0	86909.6
5 Classes	-42796.1 (104)	86300.2	86720.2
6 Classes	-42686.5 (125)	86123.0	86688.4

AIC= Akaike Information Criterion, BIC=sample size-adjusted Bayesian Information Criterion

Table S2. Socioeconomic position characteristics across identified three-class latent socioeconomic position backgrounds in 20 European countries, October 2023–April 2024 (n = 7,505)

Variable		Class 1 (%)	Class 2 (%)	Class 3 (%)
Age	18-24	0.2	28.5	0.0
	25-29	8.5	28.9	0.0
	30-39	38.0	26.0	0.0
	40-49	32.7	11.7	6.4
	50-59	17.6	3.9	7.2
	60-69	0.0	0.8	63.4
	70+	3.0	0.0	23.0
Education	I do not have a high school diploma	1.1	2.5	5.0
	Secondary education (high school or equivalent)	20.6	33.1	30.6
	Bachelor degree (university or equivalent)	30.5	38.8	26.8
	Master degree (university or equivalent)	40.2	23.8	27.6
	Phd/Doctorate	7.5	1.9	10.0
Employment	Employed	95.8	17.7	43.6
	Other	3.9	26.1	7.2
	Retired/Medical leave	0.0	2.0	46.4
	Student	0.0	37.9	0.0
	Unemployed	0.3	26.4	2.8
Perceived income	Living really comfortably on present income	2.0	22.3	7.6
	Living comfortably on present income	9.2	32.6	8.8
	Neither comfortable nor struggling on present income	35.2	33.2	23.8
	Struggling on present income	43.6	11.4	50.6
	Really struggling on present income	10.1	0.6	9.2
Migration background	Non-migrant	64.8	52.2	76.8
	First generation migrant	26.9	33.4	13.0
	Second generation migrant	8.3	14.4	10.2
